# Research Progress of Graphene-Based Flexible Humidity Sensor

**DOI:** 10.3390/s20195601

**Published:** 2020-09-30

**Authors:** Rongxuan Liang, Ansheng Luo, Zhenbang Zhang, Zhantong Li, Chongyang Han, Weibin Wu

**Affiliations:** 1 College of Engineering, South China Agricultural University, Guangzhou 510642, China; samleung@stu.scau.edu.cn (R.L.); anshengluo@stu.scau.edu.cn (A.L.); zhenbangzhang@stu.scau.edu.cn (Z.Z.); 1374487865@stu.scau.edu.cn (Z.L.); 201619030305@stu.scau.edu.cn (C.H.); 2 Division of Citrus Machinery, China Agriculture Research System, Guangzhou 510642, China

**Keywords:** graphene oxide, flexible, humidity sensors, respiratory monitoring

## Abstract

Graphene is a new type of carbon material with a flexible, two-dimensional structure. Due to the excellent stability of its lattice structure and its mechanical flexibility, graphene-based materials can be applied in flexible humidity sensors. At present, the application of graphene-based flexible humidity sensors in the fields of medical care and environmental monitoring is attracting widespread attention. In this review, the basic properties of graphene oxide (GO) and reduced graphene oxide (rGO) as moisture-sensitive materials and methods for their preparation were introduced. Moreover, three methods for improving the performance of moisture-sensitive materials were discussed. The working principle of different types of graphene-based humidity sensors were introduced. The progress in the research on graphene-based flexible humidity sensors in four respects: Human respiration, skin moisture, human sweat, and environmental humidity were discussed. Finally, the future research, following the development trends and challenges, to develop the potential of integrated, graphene-based flexible humidity sensors were discussed.

## 1. Introduction

Flexible electronic sensors are sensors made of flexible materials that can be stretched, bent, and even folded [1]. According to their different functions, flexible electronic sensors can be divided into pressure sensors, temperature sensors, humidity sensors, etc. Flexible humidity sensors have become the subject of international research due to their potential applications in bio-medicine and electronic skin.

The selection of flexible moisture-sensitive materials is the key to preparing flexible humidity sensors. In recent years, graphene has attracted a great deal of attention for various sensing applications. The excellent electrical and mechanical properties of graphene have made it a widely used material in lithium-ion batteries, supercapacitors, electronic products, and optical devices [2,3,4,5,6,7,8,9]. Because GO and rGO are rich in oxygen-containing functional groups and have a large specific surface area for molecular adsorption, they have the potential for broad application in the field of flexible humidity sensing. In fact, in addition to GO and rGO materials, polymers, and other inorganic nanomaterials have also been adapted to flexible applications, but most polymer composite materials and other types of inorganic moisture-sensitive nanomaterials are irritating to human skin. Carbon-based moisture-sensitive materials have an advantage over them in terms of bio-compatibility. In the literature on carbon-based inorganic nanomaterials published in recent years, there is a greater abundance of research results on carbon nanotubes and graphene. The advantage of graphene over carbon nanotubes is that, in the process for producing carbon nanotubes, it is easier to produce a mixture of carbon nanotubes of metal and semiconductor materials. However, it is simpler to obtain pure and high-performance GO and rGO. Moreover, when preparing moisture-sensitive films, GO and rGO sheets can be directly deposited onto a large area of flexible substrate with good dispersibility [10].

In the 1990s, researchers began to use stretchable materials with a certain degree of flexibility to prepare electronic sensors, and related research has developed significantly in recent years. In 2004, single-layer graphene was isolated for the first time. The first graphene-based gas sensor detected a single water molecule in 2007. Since that time, a number of graphene-based humidity sensors have been prepared by researchers. Capacitive and quartz crystal microbalance (QCM) humidity sensors based on a GO film coating appeared in 2011. Over the next few years, flexible humidity sensors based on graphene appeared. With the development of materials science, researchers have begun to explore humidity-sensitive materials with higher sensitivity, and have attempted to use different sensing mechanisms to prepare better-quality humidity sensors. In 2016, a multi-functional all-graphene flexible sensor capable of detecting pressure, temperature, and humidity appeared. In recent years, in order to further develop flexible devices, researchers have conferred a number of innovative functions on sensor systems, including self-powering and self-repairing functions. This was a brief timeline of the development of graphene-based flexible humidity sensors [11,12,13,14,15,16,17,18,19,20,21,22].

In this paper, we review materials and methods for the preparation of graphene-based flexible humidity sensors, and report methods for improving sensor performance. Then, according to the different sensing mechanisms, we summarize progress in the research on graphene-based flexible humidity sensors in terms of capacitance, resistance, and other mechanisms, and discuss their application and prospects in the fields of human respiration, skin moisture, human sweat, and environmental humidity.

## 2. Graphene-Based Humidity-Sensitive Materials

The core material of a humidity sensor is its humidity-sensitive material. When the humidity-sensitive material interacts with humidity (chemical action, biological action, physical adsorption, etc.), it can change the quality, thickness, and optical, mechanical, and electrochemical characteristics of the humidity-sensitive material, thereby changing the impedance between the detection electrodes. So, information about humidity can be obtained by detecting the impedance output signal.

### 2.1. Humidity-Sensitive Materials Based on Graphene Derivatives

GO is a derivative of graphene. Its structure maintains the hexagonal shape of graphene. After the oxidation process, a large number of polar oxygen-containing groups, such as hydroxyl, epoxy, and carbonyl groups, will be introduced onto the graphene layer. So, it can absorb a large number of water molecules and, with an increase in humidity, it will agglomerate into a water molecular film using the interaction between water molecules and GO materials. Due to the principle and the change in structure after the action, GO is an outstanding humidity-sensitive material that is used in humidity sensors [8,23,24,25,26,27].

GO, as a humidity-sensitive material, has many excellent properties. In terms of composition and structure, GO has good hydrophilicity due to the presence of oxygen-containing functional groups. Medheker put forward a theoretical analysis of the hydrophilicity of GO [28]. When the environment is at a different humidity, oxygen-containing functional groups will form hydrogen bonds inside and on the surface of the GO. The humidity will affect the strength and density of these interlayer hydrogen bond networks. When the hydrogen–oxygen bond distance is 2.55 Å, it can be calculated as two water molecular phases. This hydrophilic property makes it easier for GO to absorb water molecules from the environment.

Moreover, the irregular oxygen-containing region of the intermediate structure of the GO sheet, that is, the six-membered ring skeleton of carbon atoms, has hydrophobic properties, so the GO can also be dispersed in a large number of organic solvents, which demonstrates its amphiphilicity. By means of a comparison of the dispersion of GO after ultrasonic treatment and after 3 weeks of standing, GO was shown to be able to be stably dispersed in water, acetone, ethanol, ethylene glycol, and other organic solvents, which is helpful for further processing and obtaining stable GO [29]. Obtaining a high-quality and stable dispersion solution is beneficial to the production and application of moisture-sensitive film materials.

In terms of electrical properties, GO will change the dielectric constant after adsorbing water molecules. A mechanism analysis of the influence of GO on the dielectric constant of water molecules was performed by Bi’s group [30]. Under a low humidity condition, the first layer of physical adsorption of water molecules will occur on the GO membrane. Water molecules are mainly adsorbed on the GO through the double hydrogen bond. The surface-active sites (hydrophilic group, vacancy) are completed. Under the restriction of a double hydrogen bond, water molecules cannot move freely. Proton transfer between adjacent hydroxyl groups also requires a large amount of energy, so GO membranes have high resistance [31]. When the environmental humidity increases gradually, there will be many layers of water molecules on the surface of the GO film. Starting from the second layer of physical adsorption, water molecules can be physically adsorbed by a single hydrogen bond on the hydroxyl and become mobile, gradually exhibiting similar behavior to liquid water. During the process of multi-layer physical adsorption, water molecules ionize under the action of the electrostatic field to form a large number of charge carriers (H_3_O^+^). Through the Grotthuss chain reaction (H_2_O + H_3_O^+^ → H_3_O^+^ + H_2_O), proton transfer and charge transfer take place in the GO, thus reducing the resistance of the GO membrane. Additionally, there is another sensing mechanism that produces the opposite result and is worthy of discussion. When GO interacts with water molecules, GO materials can act as electron donors and exhibit p-type semiconductor properties. As the electron density increases, the number of holes in the sensing material itself decreases, so that the resistance of the sensing layer increases. In addition, when the humidity is high, the resistance of the sensitive layer increases significantly. This effect may be caused by the increase in molecular distance due to expansion and the subsequent decrease in penetration pathways [32,33,34]. Muhammad Yasin’s group studied the relationship between the electrical characteristics of GO film and the ambient temperature [35]. By drawing complex impedance spectra/Nyquist plots of GO film at different temperatures, it was found that with the increase of temperature, the impedance characteristic curve of the GO film changed more and more obviously from an approximate semicircle at low temperatures to a straight line. Because of its high impedance, GO is suitable for use as a dielectric material in the preparation of capacitive humidity sensors.

In terms of mechanical properties, due to the presence of oxygen-containing functional groups, the adsorption of water molecules from the environment will affect a GO membrane’s mechanical properties. With the increase of humidity, the elastic modulus and tensile strength of the GO membrane decrease. However, increasing the number of oxygen-containing functional groups will increase the number of hydrogen bonds, which are directly connected to the adjacent GO sheets. This will improve the elastic modulus and toughness of GO-based humidity-sensitive films, which is conducive to their application to flexible substrates [24,28,36,37].

GO can be partially reduced by the controlled removal of oxygen-containing groups. The product of the reduction is rGO. Compared with GO, it has higher electrical conductivity [8]. The structure of rGO is similar to that of graphene. However, it has some oxygen-containing groups and its production cost is relatively low. Certain methods can be used to regulate its structure and properties, so it has certain applications in the field of humidity sensing.

### 2.2. Comparison of rGO Preparation Processes and Their Influence on Sensor Performance

#### 2.2.1. Synthesis of Graphene Derivatives

At present, there are three methods for preparing GO: Hummers’ method, the Staudenmaier method, and Brodie’s method. The most commonly used preparation method is Hummers’ method [38,39,40]. The oxidants used in the Staudenmaier method and Brodie’s method need a longer time to complete the oxidation reaction, and can easily explode in the case of a high concentration. Hummers’ method uses KMnO_4_ to deoxidize graphite powder, which greatly improves the experimental safety. Moreover, rGO can be more easily and quickly prepared using Hummers’ method. In addition, this preparation method has the advantages of rapidness, simplicity, and a low preparation cost, which make industrial production easier to realize [37,41,42]. Novel GO preparation methods based on Hummers’ method and improvements to the process continue to be proposed [43,44].

The performance of rGO is related to the process for the reduction of GO, which can be reduced by many methods, such as thermal reduction, chemical reduction, and light reduction. Controlled removal of oxygen-containing functional groups on the surface of GO can change the properties and structure of the material. The thermal reduction method uses the instability of oxygen-containing functional groups on the surface of GO and can remove and form hole defects in the material through thermal annealing reduction [45].

The chemical reduction method uses chemical reagents to remove oxygen-containing functional groups on the surface of GO to achieve the reduction of GO. This method has less-stringent requirements for equipment and the environment than thermal reduction methods, and the preparation process is simpler and the cost is lower. Therefore, the chemical reduction method is suitable for use in the large-scale production of rGO.

The reduction of GO using the above-mentioned two methods requires the consumption of a large amount of energy and is not suitable for long-term development. It is also difficult to achieve the integration of flexible devices using these two methods, which limits the application of rGO in flexible electronics [31]. Photoreduction is one way to reduce GO by removing the oxygen-containing functional groups on the surface of the GO through the interaction of oxygen-containing functional groups and light. This method is a green and environmentally friendly way to reduce GO and has become the main way to reduce GO in recent years. However, the development of this method requires complicated preparation technology and expensive equipment.

#### 2.2.2. Preparation of Humidity-Sensitive Films

One of the most important components of a humidity sensor is the humidity-sensitive thin film. The commonly used methods for preparing GO composite films include titration, dip coating, spraying, spin coating, electrophoresis, Langmuir–Blodgett (L-B) membrane technology, vacuum filtration, in-situ polymerization, the sol-gel method, chemical vapor deposition, magnetron sputtering, and vacuum evaporation [40,42,46,47,48,49,50,51,52,53,54,55]. The thickness, shape, uniformity, and stability of the film and the performance of the humidity sensor will differ depending on the method used to prepare the film. Therefore, it is necessary to consider the process for the preparation of the humidity-sensitive film in advance before preparing the humidity-sensitive sensor. The advantages and disadvantages of several preparation methods are shown in Table 1.

#### 2.2.3. Methods for Improving the Performance of Humidity-Sensitive Materials

The performance of a flexible humidity sensor is closely related to the physical and chemical properties of its humidity-sensitive materials. Combined with the physical and chemical properties of humidity-sensitive materials, we can improve the performance of humidity-sensitive materials in the following three respects.

1. Controlling the morphology (structure) of the composite. Hosseini and others developed a highly sensitive flexible humidity sensor based on graphene quantum dots (QGDs) [63]. The QGDs, which were synthesized using a simple hydrothermal method, have good selectivity, a good response, a wide detection range, a short response time, a short recovery time, and a certain degree of flexibility. This sensor was used to demonstrate the application potential of QGDs in wearable electronic equipment and real-time monitoring of relative humidity (RH). Zhang and others prepared a high-sensitivity humidity sensor made of GO foam [64]. The GO foam has dielectric and direct-current (DC) conductive properties related to humidity and compression. It was found that the dielectric constant, dielectric loss, and DC conductivity all increased with the increase of RH. After compressing the GO foam, the sensitivity to humidity increased, and the maximum sensitivity to dielectric loss was more than 12 times higher than that of the DC conductivity. In addition, the dielectric properties of the GO foam enabled it to exhibit a stable and repeatable humidity response, indicating that this GO foam has great potential in the assembly of highly sensitive and lightweight humidity sensors with a repeatable humidity response.
2. Finding the best preparation conditions and methods, such as finding the most suitable annealing temperature to control the number of oxygen-containing functional groups and using the self-assembly method to make the structure stable and firm. Li prepared a flexible resistive humidity sensor based on an rGO/WS_2_ composite film [42]. It was found that the number of oxygen-containing functional groups on the surface of the composite material and the interaction between the rGO and WS_2_ were different with different annealing temperatures, which would affect the humidity sensitivity response characteristics of the sensor. Phan and others used rapid thermal annealing (RTA) to control the number of oxygen-containing functional groups in the GO [65]. Through a study on annealing at different temperatures from 400 °C to 1200 °C, it was found that with the increase of the annealing temperature, the resistance of the sample gradually decreased, and the ability of the GO to adsorb water gradually weakened. The sensitivity of the humidity sensor based on the non-annealed GO film was 35.3%. After annealing at 1200 °C, the sensitivity of the humidity sensor decreased to 0.075%, and the response time increased, but the non-annealed GO membrane was not stable in the humid environment. Therefore, although reducing the number of oxygen-containing functional groups will reduce the sensitivity of the humidity sensor, the sensitivity and stability should be considered comprehensively when designing the sensor in order to achieve a balance between them. Su’s group prepared an rGO-based flexible humidity sensor using the self-assembly method. First, they pretreated a flexible electrode with lye, then assembled the GO material layer by layer with a coupling agent, reduced the GO in situ, immersed the humidity sensor in water, and then dried it. Their experimental data show that this flexible humidity sensor has strong water resistance and its output response is unaffected and has long-term stability [66].
3. Modifying the structure of the graphene derivative according to need, such as using functional group materials to modify the GO to enhance the humidity sensitivity response, doping heteromorphic semiconductor materials to form a Schottky barrier, and preparing composite materials to improve the self-adsorption capacity and permeability structure. Su’s group modified graphene using GO as a precursor material [67,68,69]. It was found that, of the different functional groups, the amino group, the carboxyl group, and β-cyclodextrin can be used to modify GO to improve the material’s sensitivity to humidity. The amino group has relatively high activity and can easily be compared with GO. The sensor, after the reaction and modification, exhibited a good humidity sensitivity response, high sensitivity, low humidity lag, and good long-term stability. Wang’s group doped rGO with the urchinlike CuO [70]. The work function of rGO is about 4.6 ev, while that of CuO is about 5.2 ev. In a humid environment, the adsorption of water molecules reduces the Schottky barrier between rGO and CuO, thus strengthening the ion conduction strength inside the sensitive film. Compared with sensors based on the original rGO and CuO, the output sensitivity and response time of the sensors made of the composite material were improved. They all exhibited relatively good humidity sensing performance. The research group briefly explained the reasons for the increase in the impedance and the improvement in the humidity sensing performance. The water molecules adsorbed by the humidity-sensitive materials release electrons into the Schottky barrier and reduce the barrier height. This phenomenon greatly promotes the electrical conductivity of the humidity-sensitive film and improves the humidity sensing performance.

## 3. Graphene-Based Flexible Humidity Sensors

A flexible humidity sensor is generally composed of a flexible substrate, a humidity-sensitive film, and a metal detection electrode. At present, there are many kinds of graphene-based flexible humidity sensors [71,72,73]. A comparison of these sensors is shown in Table 2. The most widely used types of humidity sensors are resistive humidity sensors and capacitance humidity sensors.

### 3.1. Realization of Flexibility

**Figure 1:**
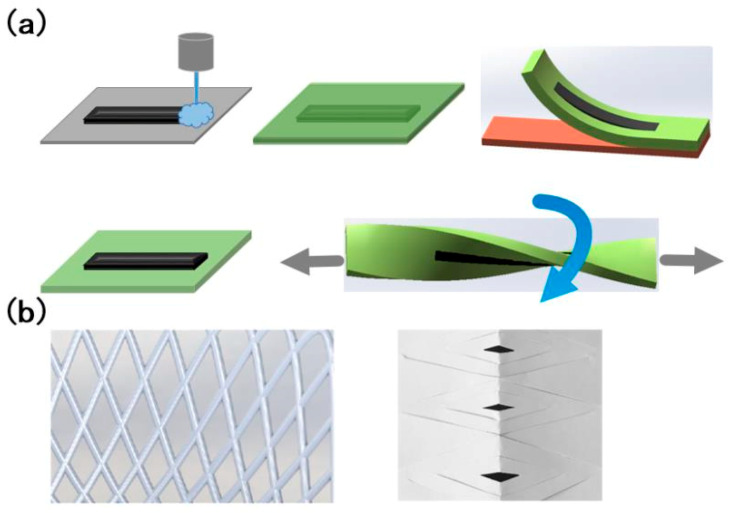
Two methods for realizing flexibility. (**a**) The flexible substrate transfer method. (**b**) The strain structure design method.

The biggest challenges for flexible humidity sensors involve their manufacture, their mechanical properties, the stability of the electrochemical performance, and their ability to maintain sensitivity after repeated bending deformations. The key to their manufacture lies in the flexibility of the electrodes/circuits. At present, the theoretical research on the design and manufacturing of graphene-based flexible electronic devices mainly focuses on the flexible substrate transfer method and the strain structure design method, as shown in Figure 1 [74,75].

The flexible substrate transfer method is a method for sequentially transferring one electrode structure to another flexible substrate. It has the advantages of strong technical compatibility and simplicity, and the operation is carried out at a normal temperature, avoiding the possibility of the temperature affecting the device’s function. Polydimethylsiloxane (PDMS) is often selected to be the flexible substrate for the transfer method. PDMS is a stretchable material that is highly transparent, chemically inert, and non-toxic. It can be applied to human skin, implanted in the body, or applied in wearable devices. In addition, polyurethane (PU), polyester resin (PET), and so on can be chosen [76]. These organic polymer materials have good flexibility and good compatibility with graphene, so they may become excellent substrates for the preparation of graphene-based flexible humidity sensors.

The strain structure design method is used mainly to provide the electrode material with a higher degree of flexibility by designing the strain structure, which can not only avoid significant deformation of the electrode material under normal working conditions, but also alleviate damage during stretching. According to the shape of the conductive layer, there are serpentine structures, paper-cut structures, three-dimensional network structures, and so on [77,78,79,80,81]. The research results on the above-mentioned methods for manufacturing flexible sensors not only provide theoretical guidance on the construction of graphene-based flexible electronic sensors, but may also further promote the innovation of related theories and technologies.

### 3.2. Sensing Mechanisms

#### 3.2.1. Resistive Type

The working principle of a resistive humidity sensor is that the moisture-sensitive film changes its impedance characteristics by adsorbing water molecules. These sensors measure the environmental humidity by changing the output electrical signal. The sensitivity of a resistive humidity sensor can be defined as:

(1) $$\mathrm{Response}=\frac{\Delta R}{R_{air}}\times 100\%=\frac{R_{humid}-R_{air}}{R_{air}}\times 100\%$$

where *R*_humidity_ is the resistance measured under humid conditions and *R*_air_ is the resistance measured under dry air conditions [32]. Resistive humidity sensors have been studied and developed in theory for a long time. They have also been mass-produced for a long time and applied in practical work. This kind of sensor deposits a humidity-sensitive film on a flexible interdigital electrode by means of a special film-forming process, which has the advantages of a simple preparation process, a simple circuit, high sensitivity, a low cost, and a small volume. Due to its excellent conductivity and easy-to-process thin films, rGO is often used in resistive-type humidity sensors.

#### 3.2.2. Capacitive Type

The working principle of a capacitive humidity sensor is that it detects the environmental humidity by changing the dielectric constant of the humidity-sensitive film through changing the transmission capacitance value. The capacitance value can be expressed by the following formula:

(2) $$C_{\mathrm{pu}}=\frac{\varepsilon_{r}\varepsilon_{0}S}{d}$$

where *C*_pu_ is the capacitance value, *S* is the effective electrode area of the capacitive sensor, *d* is the thickness of the moisture-sensitive polymer film layer, *ε*_0_ is the dielectric constant of classical vacuum, and *ε*_r_ is the dielectric constant of the moisture-sensitive polymer material. The structure of a capacitive flexible humidity sensor is similar to that of the resistance type, and the dielectric layer is covered on two of the flexible cross finger electrodes. At present, most humidity sensors use capacitance sensing technology [82,83]. This kind of humidity sensor is very sensitive to changes in humidity, and has the advantages of low power consumption, a high output signal, a short response time, a small temperature coefficient, and great product interchangeability, so it is widely used in practice. Compared with resistance humidity sensors, high-resistance GO is more suitable for use as a dielectric layer in the preparation of capacitive humidity sensors.

#### 3.2.3. Other Types of Graphene-Based Flexible Humidity Sensors

**Figure 2:**
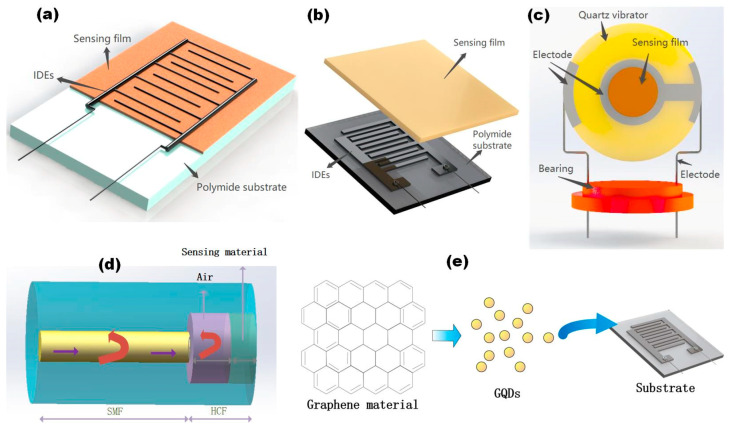
Different types of humidity sensors [71,72,73]. (**a**) Resistive humidity sensor. (**b**) Capacitance humidity sensor. (**c**) Quartz crystal microbalance (QCM) humidity sensor. (**d**) Fiber-optic humidity sensor. (**e**) GQDs material humidity sensor.

In addition to resistive flexible humidity sensors and capacitive flexible humidity sensors, there are also high-performance humidity sensors based on other technologies and instruments, such as optical fiber humidity sensors and QCM humidity sensors. In addition to GO-based and rGO-based humidity sensors, graphene derivatives, such as graphene quantum dots prepared from GO, can be used to prepare flexible humidity sensors. GQDs have also been combined with optical fiber technology to prepare a new type of humidity sensor with higher sensitivity [73]. The sensors mentioned above is shown in Figure 2, which have their own advantages and specific application conditions [71].

1. The humidity sensing mechanism of a fiber-optic humidity sensor is that the properties of refracted or reflected light waves are changed after the moisture-sensitive material adsorbs water molecules. The changes in properties can be detected by the amplitude, polarization amplitude, frequency shift, or phase shift of the light waves. Because of their small volume and light weight, optical fiber sensors have low transmission loss and a strong multiplexing ability and can also realize multi-parameter and long-distance detection. Because of their excellent corrosion resistance and anti-electromagnetic interference ability, optical fiber sensors are suitable for use under strong magnetic conditions and in harsh environments [84,85,86].
2. QCM is a non-destructive technology. The sensing mechanism of a QCM humidity sensor is to coat a layer of humidity-sensitive film on the electrode. After the moisture-sensitive material is deposited on the electrode, Sauerbrey’s equation can be used to convert the dynamic adsorption mass into a resonance frequency shift.
  (3) $$f=-\frac{2f_{0}{}^{2}}{A\sqrt{\rho u}}\Delta m$$
  where *f*_0_ is the resonance frequency of the QCM, *A* is the effective area, and *ρ* and *μ* are the density and shear modulus of the quartz crystal, respectively [87,88]. Compared with the traditional humidity sensors, QCM humidity sensors have the characteristics of a small size, a high frequency, and intelligence.
3. GQD material is an important graphene derivative whose sheet size is smaller than 100 nm, and it has a quantum confinement electron state. GQD material has excellent hydrophilicity, a large specific surface area, and a small sheet size [89,90]. The film, which is formed by stacking, has a large number of voids, which enable water molecules to penetrate into the inside of the humidity-sensitive thin film more quickly and accelerate the sensor’s humidity-sensitive response. It has received a great deal of attention as a new type of moisture-sensitive material.

**Table 2:** Graphene-based sensor performance and comparison.

Sensor Type	Sensitive Material	Preparation Method	Measurement Range	Sensitivity	Response Time	Reference
Resistive type	G/methyl red M-R	Ink jet printing	5–95%	96.36% (∆*R*/R)	0.25 s	[91]
Resistive type	G/PEDOT:PSS/PI	Ink jet printing	31–95%	40% (∆*R*/R)	20 s	[92]
Resistive type	PEDOT: rGO-PEI/Au	Ink jet printing	11–98%	51.6% (∆*R*/R)	20 s	[93]
Capacitive type	GO/paper	Self-assembly	30–90%	38% (Δ*C*/C_0_)	Not given	[94]
Capacitive type	GO/PEDOT:PSS	Sedimentary method	Not given	1220 pF/%RH	Not given	[95]
Capacitive type	GO/Ag	Drop coating	11~97%	25809 pF/%RH	8 s	[42]
Optical type	rGO	self-assembly	50.5~85%	–4.118 dB/%RH	Not given	[96]
QCM type	rGO/PEO	Layering	11.3 to 84%	20 Hz/%RH	11 s	[97]
GQD type	GQD/PI	Drop-casting	1~100%	(~390 for a RH change of 99%)	12 s	[63]

## 4. Applications of Graphene-Based Flexible Humidity Sensors

**Figure 3:**
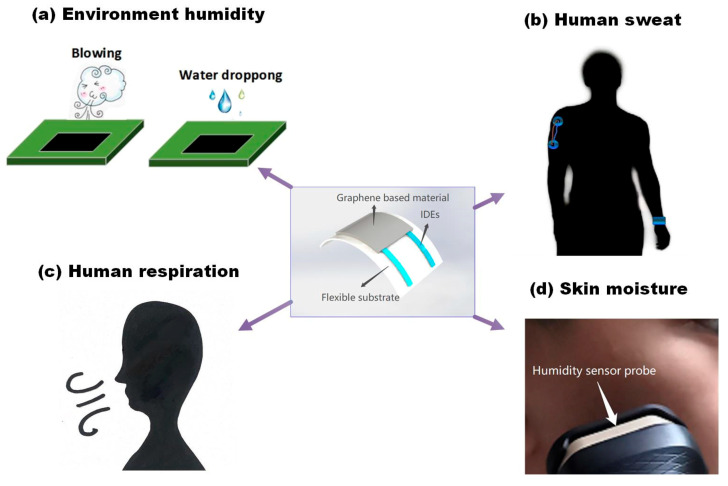
The applications of graphene-based humidity sensors.

Due to their high sensitivity, excellent flexibility, good stretchability, and stability, graphene-based flexible humidity sensors have great potential for application in such fields as electronic skin, personal health monitoring, and wearable and stretchable humidity sensors [98,99,100,101]. They can be placed on the human body or clothes to detect signals from human activity and obtain various kinds of physiological information according to the object of implementation. As shown in Figure 3, the applications of graphene-based flexible humidity sensors can be classified into four categories: Monitoring human respiration, monitoring skin moisture, detecting sweat, and detecting environmental humidity.

### 4.1. Human Respiration

Respiratory rate can be considered to be a key indicator of human health, and monitoring changes in an individual’s respiratory rate and depth of breath can be used in medical diagnosis [102,103]. Traditional breath monitoring instruments have the disadvantages of a large volume, a high production cost, and using rigid materials as substrates, which leads to an inability to fold and poor portability. However, due to their mechanical toughness, large specific surface area, and high conductivity, graphene-based flexible humidity sensors can provide excellent humidity sensing performance in the field of respiratory monitoring, have a certain degree of flexibility and a low cost, and can make up for the shortcomings of traditional rigid respiration monitoring instruments. To date, a number of studies on graphene-based flexible humidity sensors for application in this field have been performed. Ye’s team prepared a GO humidity sensor that can self-supply energy by means of ink-jet printing [104]. By using the humidity-sensitive characteristics of GO material after spontaneous polarization, the device’s structural parameters were optimized, and a humidity sensor with excellent sensitivity, rapid response and recovery times, multiple circulation stability, and long-term aging stability was obtained. Based on this sensor, the detection of different respiratory frequencies, such as normal static respiration and rapid respiration, in the human body can be realized. Moreover, the humidity sensor is simple to prepare, low in cost, not easily interfered with by human actions and the external environment, and has practical value.

### 4.2. Skin Moisture

The moisture content of human skin is also a key indicator of human health. Graphene-based flexible humidity sensors can detect skin moisture and show great potential for use in wearable devices suitable for evaluating moisturizing products. At present, there are few research results on this kind of sensor. On the one hand, for contact sensor equipment, when in contact with skin, the sensor needs to have a certain degree of flexibility, a better fit for the skin, and be harmless to the skin to prevent damage with long-term use. On the other hand, the preparation of non-contact sensor equipment needs theoretical guidance. Graphene-based flexible humidity sensors for the detection of moisture and humidity in the skin have yet to be developed. However, researchers have attempted to establish a functional relationship between the sensitivity of PIM-based sensors and the moisture content of human skin and developed high-precision skin moisture measurement instruments that may provide a solution for graphene-based flexible humidity sensors [101].

### 4.3. Human Sweat

There are many ions in human sweat. The composition and content of ions in human sweat are also key indicators of human health. It is very important to personal health monitoring and exercise monitoring to sample and analyze fresh sweat using sensors. At present, there are many types of studies on the implementation of personalized health monitoring by means of wearable sensor technology. The monitoring of ions in sweat requires the wearable sensor to capture fresh sweat. Guijun Li and others reported on the development of wearable sweat capture devices using patterned graphene arrays with controllable wetness and conductivity for the simultaneous capture and electrochemical measurement of sweat droplets [99]. Sweat droplets showed strong adhesion to and moderate movement on the super-hydrophilic patterned graphene arrays. These sensors can be used for personalized, whole-body, and real-time monitoring of sweat for the purpose of sports performance optimization, and physiological health tracking.

### 4.4. Ambient Humidity

Life depends on environmental moisture. Life needs to sense the humidity and temperature of the outside world and provide feedback. As an important organ for sensing the outside world, the skin needs to be repaired by electronic equipment and medical means if it is damaged or even necrotic. Electronic skin is a system that can simulate human skin, sense the external pressure, temperature, and humidity, obtain other information, and provide feedback, which also requires the integration of flexible humidity sensors in the electronic skin. Most sensors that can sense pressure, temperature, and humidity are rigid and cannot perform synchronous monitoring. Recently, a multifunctional sensor was prepared by means of spraying a mixture of carbon black (CB) and rGO on a paper substrate [105]. It can detect external strain, humidity, temperature, and pressure with a single device and has high sensitivity. In addition, the sensor is easily degraded in water, but it can be reused after drying, which illustrates its strong stability.

In general, graphene-based flexible humidity sensors are widely used in the field of respiratory monitoring. Moreover, the technology for their preparation is becoming increasingly simple, their cost is gradually being reduced, and their performance is constantly being improved, which should also promote the development of wearable medical systems.

## 5. Summary and Outlook

Due to its large specific surface area and the large number of hydrophilic oxygen-containing functional groups, GO has an excellent capacity for water absorption. GO and rGO are two common humidity-sensitive materials. By controlling and optimizing these materials’ structure, improving the preparation conditions and methods, or directly modifying them, the sensing performance of the sensor can be improved. In addition, GQD material has a small sheet size that enables a film formed by stacking to contain a large number of voids, which enable water molecules to penetrate into the humidity-sensitive film more quickly and accelerate the humidity-sensitive response of the sensor. GQD material is a graphene-based material with the potential for broad application in humidity-sensitive materials.

The large-scale production of high-quality humidity-sensitive materials is a basic premise of the application of graphene-based flexible humidity sensors. The existing spin-coating and inkjet printing technologies can be used to realize the preparation of low-cost humidity-sensitive films [104,106,107]. These two technologies are simple to operate, but the control precision needs to be improved. The development of manufacturing technologies with a higher control accuracy will be beneficial to the large-scale production of graphene-based flexible humidity sensors.

In the process of preparing graphene-based flexible sensors, the flexible substrate transfer method and the stress structure design method can be used to obtain a high degree of flexibility. Different graphene-based materials have different sensing mechanisms. QCM sensors and fiber-optic sensors use QCM sensing devices and fiber-optic sensing technologies, respectively. As new types of sensors that integrate multiple technologies, they have excellent humidity sensing characteristics, including a short response time, low humidity hysteresis, and high sensitivity. In addition, these two new types of flexible humidity sensors have high repeatability, good long-term stability, and a long life. They have very broad future research prospects.

Finally, the actual application of graphene-based flexible humidity sensors in human respiratory monitoring, skin moisture detection, sweat analysis, and environmental humidity detection requires us to integrate sensor technology with other technologies. Currently, the main problem of multi-function sensors is that the recognition accuracy between multiple signals still needs to be improved. Sensor materials need to have a high degree of stability in order for us to complete the integration of data transmission and processing units and ultimately achieve the goals of minimizing equipment size and optimizing performance.

## Figures and Tables

**Table 1:** Comparison of processes for the preparation of graphene oxide (GO) humidity-sensitive films.

Preparation Method	Advantages	Shortcomings	Reference
Spraying method	Simple, convenient, a high degree of orientation, and large-scale production.	The resistance is limited by the film’s thickness.	[42,56,57]
Magnetron sputtering	High accuracy, an unlimited preparation area, a low preparation temperature, and a simple process.	A complex operation and uses expensive machines.	[42,58]
Sol-gel method	Good uniformity, strong operability, and makes it easy to realize large-scale production.	A complex operation, a long preparation cycle, and it is difficult to manipulate the ordered assembly of graphene sheets.	[59]
Chemical vapor deposition	High quality, good crystallinity, and few defects. The film can be deposited on large areas.	The thickness is limited by the substrate, and this method matches poorly with the device manufacturing process.	[60,61]
Vacuum filtration	Mature, simple, and improves the orientation of lamellar.	Time-consuming, the film is too thick, which causes surface wrinkles, and the size is limited by the size of the filter membrane.	[56]
Self-assembly	A simple process. The film has a firm structure and uniform thickness.	The membrane is too dense and flat to adsorb and desorb water. The size is limited by the substrate and equipment. Difficult to use to carry out mass production.	[60]
Inkjet printing	Controllable thickness and improves the rate of utilization of raw material.	The control accuracy of the print head and printing system is insufficiently high.	[62]
